# Gaze behavior when looking at paintings may predict autistic traits

**DOI:** 10.1002/pchj.810

**Published:** 2025-01-06

**Authors:** Yizhen Zhou, Mana Nishimura, Hideaki Kawabata

**Affiliations:** ^1^ Global Research Institute Keio University Tokyo Japan; ^2^ Department of Psychology Keio University Tokyo Japan

**Keywords:** artwork, eye tracking, gaze behavior, social attention

## Abstract

From infancy, we spend considerable time absorbing social information from the external world. Social information processing, which starts with looking at facial expressions, affects behavior and cognition. Previous research has demonstrated that looking behaviors at social cues such as faces may differ in individuals with autism spectrum disorder (ASD) by using eye‐tracking studies with real photographs and movies. However, mixed results have been reported. In this study, we examined whether autistic traits in adults affected gaze behavior when participants viewed paintings. The eye‐tracking results indicate that gaze patterns change over time during a 20‐s free‐viewing task. Although the fixations were not influenced during the first 10 s of the viewing, autistic tendencies affected gaze behavior after the overview of the painting was completed: the higher the autism‐spectrum quotient scores, the shorter the fixation duration and the fewer the fixations on the facial areas of the paintings during the latter 10 s of viewing time. This result indicates that the atypical gaze behavior was more likely to be modulated by a generalized attentional process for endogenous orienting with reduced interest in social cues. Gaze patterns of viewing paintings may be used to predict autistic tendencies among people undiagnosed but suspected of having ASD.

## INTRODUCTION

Since infancy, we have spent a significant amount of time interacting with the external world to acquire social information. We often have a preference for social cues such as facial expressions because they are essential for the development of communication and social skills (Gliga & Csibra, 2007; Vuilleumier, 2002). However, individuals with autism spectrum disorder (ASD) can find processing these social cues difficult (American Psychiatric Association, 2013). Recent studies found that individuals with ASD have deficits in social skills (Uljarevic & Hamilton, 2013; Weigelt et al., 2012), which may result from atypical attention allocation or reduced attention to social stimuli (Falck‐Ytter & von Hofsten, 2010). The atypical attention processes in individuals with ASD have been investigated using eye‐tracking technology, a noninvasive technique that is widely used to track eye movements by measuring the position of the gaze point or the eye's movement relative to the head. Previous eye‐tracking studies have found a correlation between reduced attention to social stimuli, increased attention to nonsocial stimuli, and autistic behavior (Bird et al., 2011; Chawarska et al., 2012; Klin et al., 2002; Shic et al., 2011). Among all social stimuli, faces play a vital role in social interactions. Faces convey information that is essential to evaluate others' intentions and mental states (Jack & Schyns, 2015).

Although visual attention to faces in individuals with ASD has been intensively studied using eye‐tracking techniques, research has yielded mixed results. Some studies have found reduced attention to faces among individuals with ASD, while others reported no difference between participants with or without ASD. For instance, Fletcher‐Watson et al. (2009) demonstrated that individuals with ASD paid less attention to faces when shown photographs of people. A similar trend was also observed by showing clips of movies to groups of people with ASD (Rice et al., 2012). Contrastingly, a nonsignificant difference in attention patterns between ASD and typical development (TD) groups has also been reported in studies using static images and videos (Birmingham et al., 2011; Gastgeb et al., 2011; Kuhn et al., 2010).

Yet, no clear picture has emerged regarding whether the development of ASD is fundamentally linked to social attention (Falck‐Ytter et al., 2023). If a specialized social attention process exists and the atypical social attention behaviors are fundamentally related to ASD, the mechanism of face orienting should differ among individuals with ASD even when the complexity of social contexts is moderate. Thus, it is necessary to examine gaze patterns when the “social” scenes are not visually complex and dynamic. However, most eye‐tracking studies have used naturalistic contexts of real scenes and people that contain complex information, in order to examine the attention processes in ASD individuals toward social cues. The backgrounds of the scenes, for instance, are not directly related to social stimuli. Therefore, it may be difficult for individuals with ASD to handle this extraneous information. A few studies have used drawings or cartographic images to reduce ecological validity and found that a specialized social attention process seems unlikely. For instance, Kemner et al. (2007) found that the fixation numbers and durations of both ASD and TD children were larger and longer for cartoon‐like drawings of complex objects than for simple ones, while no difference between the two participant groups was found. However, in terms of ecological validity, cartoons used in previous studies may have been overly simplistic or exaggerated. For example, the eyes in cartoons are typically large. Uncertainty remains regarding whether the preferential looking to social stimuli is atypical in individuals with ASD.

**FIGURE 1:**
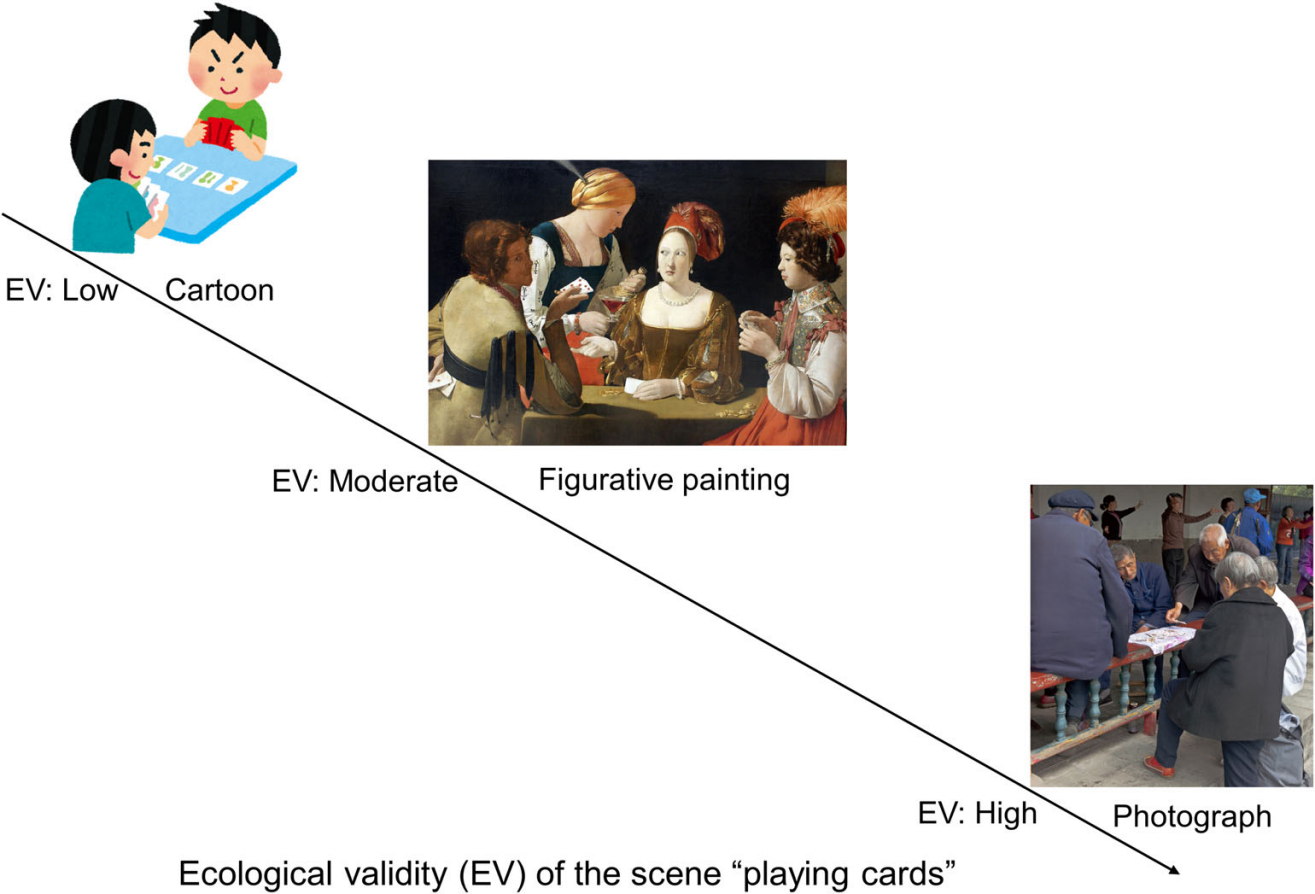
The scene of “playing cards” represented by a cartoon, a figurative painting, and a photograph. The images used in the figure are copyright‐free images (the photograph "A group of senior citizens playing cards on a railing at Temple of Heaven Park in Beijing" is credited to Daniel Case, used under CC BY‐SA 3.0 [https://commons.wikimedia.org/wiki/File:Old_people_playing_cards_at_Temple_of_Heaven_Park,_Beijing.jpg]).

Therefore, it is necessary to verify whether the gaze deficit on social stimuli can be found at a moderate level of ecological validity, for instance, when observing paintings. Paintings differ from cartoons in terms of “simplicity,” as they convey more complex narratives using intricate details and textures. Figurative paintings are more concrete than cartoons and reflect real scenes in an artistic way. Figurative paintings are not identical to real scenes as they are the result of painters' imaginations; however, unlike cartoons, they mimic what the human eye sees while filtering extraneous information, to emphasize the narrative or emotional aspects of the main subject without distractions. Compared with paintings, the background information in real scenes may be defined as “extraneous” because such information in paintings is often eliminated or simplified. In the real scenes represented by photographs or movies, the environment or the texture of the objects in the environment is vividly reflected. They reflect reality in the way they capture light and create visual representations of a moment in time. In painting creation, the background may also be omitted to improve the painting's composition for balance, harmony, or visual effect. Thus, paintings have a moderate ecological validity (see the example of depicting the “playing cards” scene in Figure 1).

Moreover, it is particularly intriguing to investigate gaze behavior during aesthetic appreciation, such as when viewing paintings. Aesthetic perception is a human prerogative. It is crucial for many aspects of human cognition, including emotion and social cohesion (Wassiliwizky & Menninghaus, 2021). In recent years, research on aesthetics, including the perception of paintings, has grown rapidly. Studies reveal that ASD individuals' capability of aesthetic perception is retained at an implicit level of eye‐tracking, although they showed impairment in subjective aesthetic judgment at an explicit level (Mazza et al., 2020). Previous studies on assessing gaze behavior when viewing paintings demonstrated a preference toward social information and that the faces of human subjects in figurative paintings caught viewers' attention (Klein et al., 2014; Massaro et al., 2012). Although people may look at different parts of the painting, they all fixated on faces during the free‐viewing task (Zhou et al., 2024). Importantly, gaze patterns may also change over time (Heidenreich & Turano, 2011; Zhou et al., 2024). For example, the participants' mean fixation durations (MFDs) on paintings increased as the viewing time progressed. At the beginning of the viewing period, participants obtained an overview of the entire painting. Then, they progressed to the stage of aesthetic judgment. Sufficient time to obtain an overview is considered to be 10 s (Locher et al., 2007), while the median looking time at a painting in a real art museum was 17 s (Smith & Smith, 2001). For this reason, we defined a presentation time of 20 s for each painting. We investigated the visual attention at the first and second segments (i.e., 0–10 s and 10–20 s) of the viewing period separately. The decreased visual attention to faces during either the first or both segments of the 20 s may be interpreted as an atypical social behavior, induced by an impairment of a specialized attention process for orienting faces. Contrastingly, decreased visual attention during the second segment may hint at a general attentional process if no difference was observed during the initial processing for the overview of the paintings. In other words, autistic traits do not lead to an automatic avoidance of faces, but a reduced interest in them.

More interestingly, using paintings as stimuli might help understand the influence of autistic traits on eye movements in cultural contexts, considering that in realistic social scenes, cultural expectations may influence gaze behavior. For instance, in Japan, people often avoid direct eye contact due to cultural expectations. When making direct eye contact, Japanese participants perceive direct gaze features as more unapproachable, unpleasant, and angry (Akechi et al., 2013). Traditionally, Japanese children are even instructed to focus on the necks of other people, instead of their faces (Morsbach, 1973). Therefore, in realistic scenes, a behavior bias may exist regardless of autistic traits. Thus, the robustness of the association between gaze deficits and autistic tendencies on visual attention may be assessed by figurative paintings, as they would not be subject to the same cultural restrictions as naturalistic contexts.

Instead of examining individuals with ASD, this study assessed gaze patterns when looking at paintings among undiagnosed people with different levels of autistic traits. Recent surveys have shown that the number of individuals undiagnosed but suspected of having developmental disabilities is substantial in Japan among university students (Japan Student Services Organization, 2018). Given the cultural contexts in which many autistic behaviors are slightly exaggerated versions of behaviors commonly observed in Japanese society (Atherton et al., 2023), it is worthwhile to investigate the relationship between gaze behavior and autistic traits among the general population. Among the autistic population, the prevalence of men with ASD is evident (Loomes et al., 2017). However, girls who meet the criteria for ASD are at a disproportionate risk of not receiving a clinical diagnosis, as results of previous studies have varied significantly and substantially. Therefore, the effect of gender should also be investigated among the general population.

Additionally, previous research has demonstrated that biological stimuli, such as human features and bodies, are processed differently than artifactual ones. Biological stimuli, especially faces, are more easily remembered (Kapsetaki & Zeki, 2022). If individuals with high autistic traits have a specialized process of innate face preference that leads to an avoidance of the facial areas of the characters in the paintings, their memory of the paintings might be influenced.

This study investigated whether visual attention to figurative paintings varied according to autistic trait scores using a free‐viewing task. The autistic trait scores of all participants were measured using the autism‐spectrum quotient (AQ) Japanese version (Wakabayashi et al., 2004) translated from the AQ (Baron‐Cohen et al., 2001). Every painting viewed by the participants realistically depicted multiple human characters. We selected facial areas and body parts in the paintings as areas of interest (AOIs) and compared eye‐tracking measures among participants for these AOIs. We examined the temporal aspect of gaze patterns to determine whether the different looking behaviors were caused by a domain‐specific attentional process or a generally reduced interest in social cues. The difference in eye‐tracking measures according to autistic trait scores would provide some support for the hypotheses that the atypical attention process associated with higher autistic tendencies is independent of ecological validity. In addition, a recall task was conducted after the participants had finished the free‐viewing task to investigate whether autistic traits influenced the memory of the paintings. Before the experiment, the participants were only informed that they would view the paintings but were not instructed to remember the paintings they saw.

## METHOD

### Participants

A total of 46 participants in the age range of 19–55 years (*mean* = 23.1 years; standard deviation [*SD*] = 6.82 years; 33 women) took part in the eye‐tracking experiment and recall task. None of the participants had experience with art criticism, and they all had normal or corrected‐to‐normal vision. This study was approved by the Ethics Committee of Keio University and conducted in line with the 2013 Declaration of Helsinki (approval number: 22016). Prior to the experiment, all participants provided written informed consent.

### Materials

#### Stimuli

We selected 50 figurative paintings as target stimuli (Table S1 shows a complete list of paintings). All paintings were displayed on a light gray background. The paintings were rescaled to approximately 905 pixels in height while maintaining the original aspect ratio; as a result, the widths varied between 528 and 1899 pixels.

#### Apparatus

The experiment was performed in a semidark and quiet room. Tobii Pro Spectrum, a screen‐based eye tracker manufactured by Tobii AB with a sampling frequency of 300 Hz and 9‐point calibration/validation, was used to track both eyes. All stimuli were displayed on a 23.8″ In‐Plane Switching monitor (FlexScan EV245, EIZO Corporation) positioned 63 cm away from the eyes. The participants placed their chin on a chin rest to stabilize their heads. We utilized a toolbox named Titta (Niehorster et al., 2020) to create and run stimuli presentations. This toolbox enabled direct control of the eye tracker from Python, whereas eye‐tracking data were collected using the Tobii Pro Lab software provided by Tobii AB.

### Procedure

**FIGURE 2:**
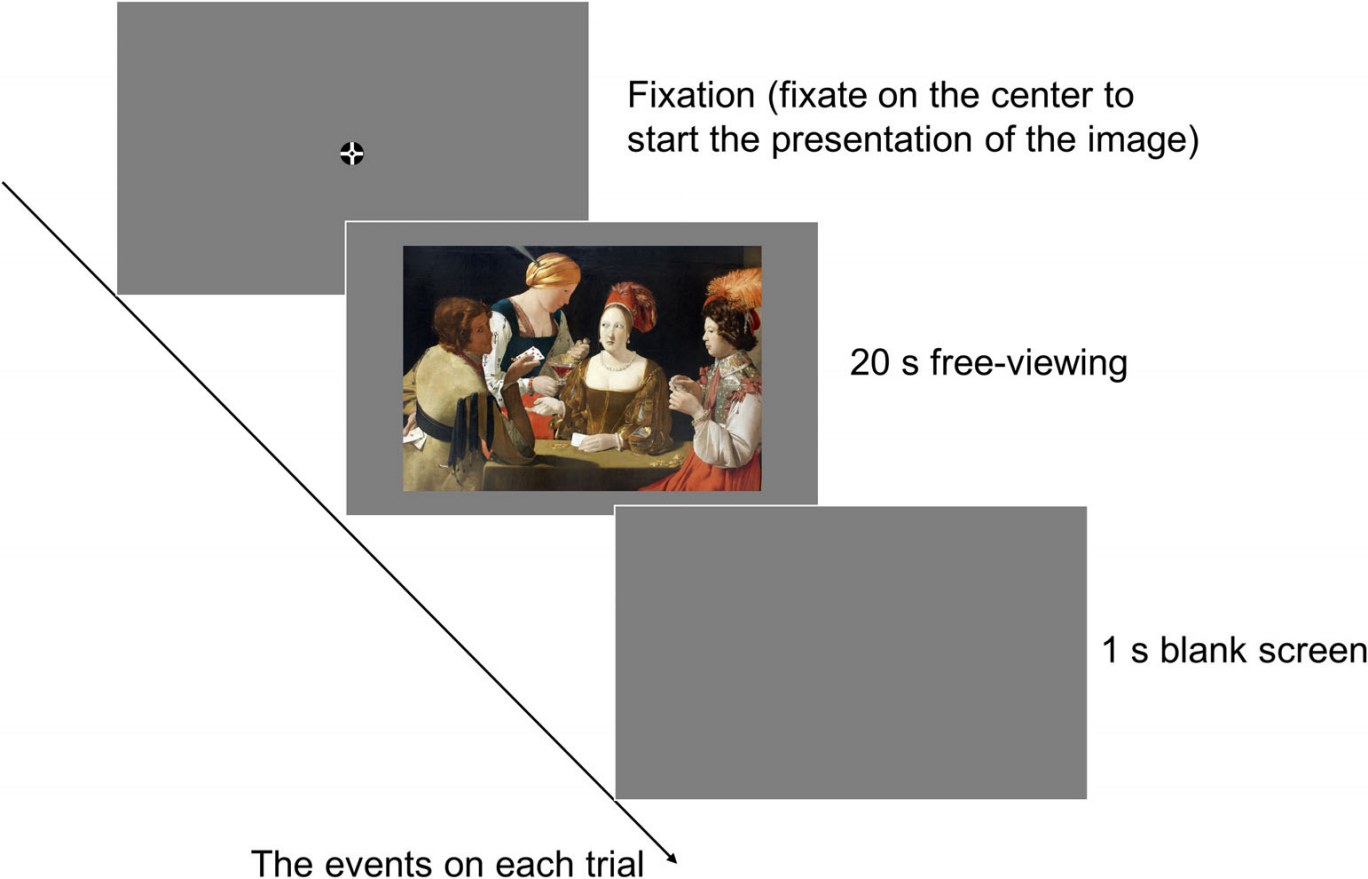
A schematic representation of the events on each trial.

The experiment included two tasks: a free‐viewing task and a recall task. In the free‐viewing task (see Figure 2), participants viewed 50 figurative paintings freely. Before the task, participants calibrated the eye tracker. They were also asked to complete two practice trials with two figurative paintings that would not be presented again for the rest of the free‐viewing task. All stimuli were presented in random order, with each painting shown on the screen for 20 s. Before each trial, a fixation cross appeared at the center of the screen, and participants had to fixate on it to proceed to the subsequent trial. If this fixation was unsuccessful, the participants were instructed to recalibrate. Participants were also given breaks after every 25 stimuli, and a recalibration of the eye tracker was necessary after the break. It took each participant about 25 min to complete the entire task.

Following the free‐viewing task, participants were required to complete a recall task of all paintings mixed with another set of 50 new paintings that had not been presented in the free‐viewing task. The same monitor used in the free‐viewing task was used for the recall task. All paintings were presented in random order. Participants were asked whether the target painting had been presented in the previous free‐viewing task. Once the participants had finished the recall task, they were required to complete the AQ questionnaire mixed with pseudo‐questions taken from the Japanese version of the Ten‐Item Personality Inventory (Oshio et al., 2012).

### Data analysis

We analyzed all 2300 trials collected from 46 participants in the free‐viewing task. Each participant's fixation metrics were obtained via the Tobii Pro Lab software. The fixations were detected using Tobii's velocity‐threshold identification fixation filter (see Komogortsev et al., 2010; Salvucci & Goldberg, 2000). We selected the facial areas and body parts of human characters on every painting as our AOI and compared the differences between all areas of the paintings. First, we compared group means of the eye‐tracking measures in terms of total fixation duration (TFD), fixation count (FC), and MFD between the first 10‐s and the second 10‐s viewing period. Second, a linear mixed model (LMM) analysis was conducted on fixations. The LMMs were generated using the lmer function from the R package lmerTest (Kuznetsova et al., 2017). TFD, along with FC and MFD, was used as the dependent variable.

**FIGURE 3:**
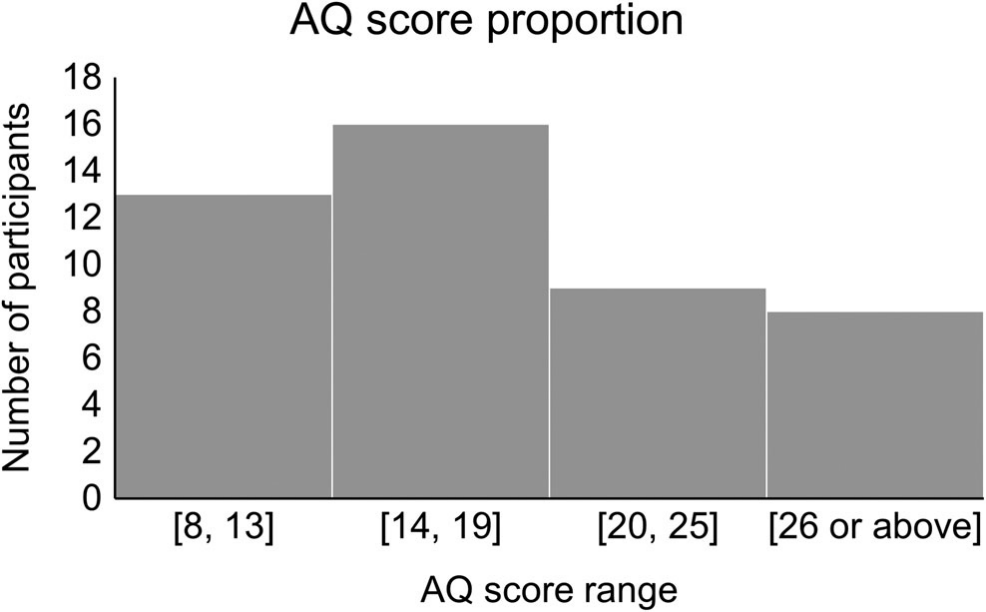
The autism‐spectrum quotient (AQ) score proportion of the participants.

For the recall task, we ran a generalized linear mixed model (GLMM) analysis of participants' correct or incorrect responses to the target painting using the glmer function in the lmerTest package (Kuznetsova et al., 2017). All data generated and analyzed in this study can be found in the Supplementary Material files. The proportion of the AQ scores is shown in Figure 3, and the scores of each participant can be found in Table S2.

## RESULTS

### Free‐viewing task

**FIGURE 4:**
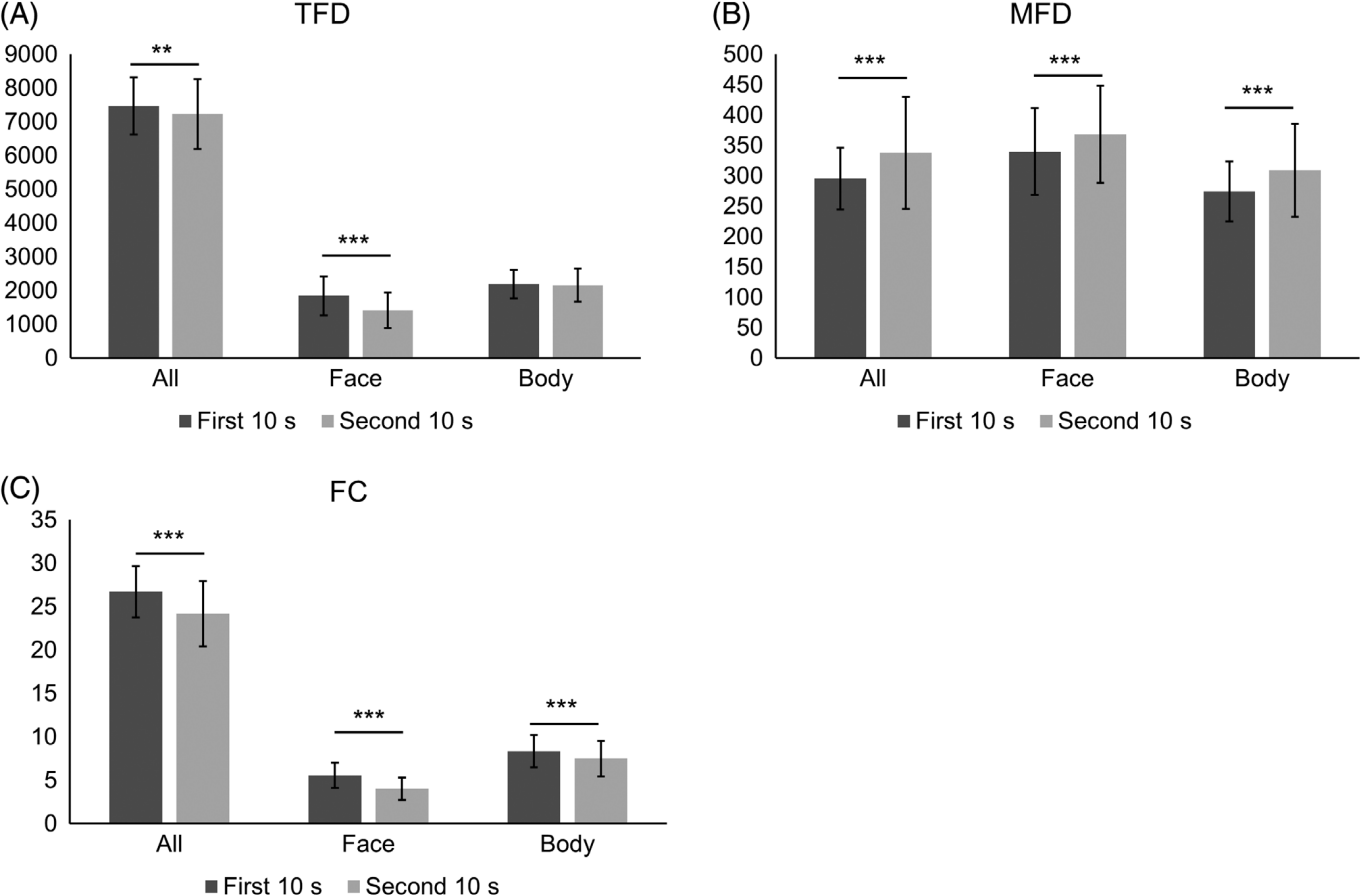
The group means of (A) total fixation duration (TFD), (B) mean fixation duration (MFD), and (C) fixation count (FC) during two viewing periods, respectively. Error bars represent the standard errors of the mean. **p < .01, ***p < .001.

Table 1 indicates the overall group means of each eye‐tracking measure in terms of TFD, MFD, and FC during the 20 s viewing time. Figure 4 shows the TFD, MFD, and FC group means during each viewing period. A paired‐samples *t*‐test was conducted to confirm whether there was a difference between the two viewing periods. As expected, the gaze patterns changed over time. We found that the TFDs on the whole painting and facial areas, but not on body parts, significantly reduced in the second viewing period (whole painting: *t* [45] = 2.97, 95% CI [0.13, 0.74], *p* = .005; face: *t* [45] = 11.66, 95% CI [1.26, 2.17], *p* < .001; body: *t* [45] = 0.82, 95% CI [−0.17, 0.41], *p* = .415). In contrast, the MFDs increased significantly during the second viewing period (whole painting: *t* [45] = −4.41, 95% CI [−0.97, −0.33], *p* < .001; face: *t* [45] = −5.18, 95% CI [−1.09, −0.43], *p* < .001; body: *t* [45] = −4.35, 95% CI [−0.96, −0.32], *p* < .001), while the number of fixations reduced significantly (whole painting: *t* [45] = 8.24, 95% CI [0.83, 1.60], *p* < .001; face: *t* [45] = 14.1, 95% CI [1.56, 2.59], *p* < .001; body: *t* [45] = 6.00, 95% CI [0.54, 1.22], *p* < .001).

**TABLE 1:** Overall group means and standard deviations of eye‐tracking measures for fixation durations and count.

	TFD	MFD	FC
Whole painting	14,694.70 ± 1813.04	316.52 ± 66.72	50.90 ± 6.47
AOI (face)	3260.82 ± 1079.82	349.49 ± 70.31	9.61 ± 2.65
AOI (body)	4346.23 ± 870.80	287.56 ± 54.18	15.83 ± 3.79

Abbreviations: AOI, area of interest; FC, fixation count; MFD, mean fixation duration; TFD, total fixation duration.

The LMM analysis was conducted to examine the fixed effects of the autistic trait scores (which served as a continuous fixed effect) and the gender of the participants (as a categorical fixed effect) in the free‐viewing task. A full model including the fixed effects of both the autistic trait scores and the gender of participants was constructed. We then compared the full model with the reduced models without the fixed effects to determine whether the effects were significant. We also included the interaction between autistic trait scores and gender to evaluate whether the relationship between visual attention and autistic trait scores differed between women and men. Random intercepts of painting and participant were included in the model to account for the dependency of observations.

For the overall TFD, MFD, and FC, no significant interaction between autistic trait scores or gender was found during the first 10‐s viewing period. A likelihood ratio test compared the full model and the reduced model without the interaction: whole painting ([TFD; *X* ^2^ = 1.97, *p* = .16], [MFD; *X* ^2^ = 0.18, *p* = .67], [FC; *X* ^2^ = 0.019, *p* = .89]), face ([TFD; *X* ^2^ = 0.61, *p* = .43], [MFD; *X* ^2^ = 0.30, *p* = .58], [FC; *X* ^2^ = 0.042, *p* = .84]), and body ([TFD; *X* ^2^ = 0.031, *p* = .86], [MFD; *X* ^2^ = 0.13, *p* = .72], [FC; *X* ^2^ = 0.18, *p* = .67]). There was no significant effect of autistic trait scores or gender on the AOIs of the whole painting, face, and body for the TFD, MFD, and FC (see Tables 2 and 3 for the summary of the results).

**TABLE 2:** Summary of the influence of autistic trait scores on eye‐tracking measures (the first 10‐s viewing period).

	TFD		MFD		FC	
	*X* ^2^	*p*	*X* ^2^	*p*	*X* ^2^	*p*
Whole painting	2.75	.097	0.0008	.98	2.25	.13
AOI (face)	2.44	.12	0.1	.75	3.56	.6
AOI (body)	0.083	.77	0.013	.91	0.0019	.97

Abbreviations: AOI, area of interest; FC, fixation count; MFD, mean fixation duration; TFD, total fixation duration.

**TABLE 3:** Summary of the influence of gender (men) on eye‐tracking measures (the first 10‐s viewing period).

	TFD		MFD		FC	
	*X* ^2^	*p*	*X* ^2^	*p*	*X* ^2^	*p*
Whole painting	2.49	.11	2.14	.14	0.14	.71
AOI (face)	3.28	.07	1.69	.19	1.96	.16
AOI (body)	0.094	.76	0.041	.84	0.0	.99

Abbreviations: AOI, area of interest; FC, fixation count; MFD, mean fixation duration; TFD, total fixation duration.

Similarly, there was no interaction between autistic trait scores and gender during the second 10‐s viewing period: whole painting ([TFD; *X* ^2^ = 0.033, *p* = .86], [MFD; *X* ^2^ = 0.35, *p* = .56], [FC; *X* ^2^ = 0.50, *p* = .48]), face ([TFD; *X* ^2^ = 0.33, *p* = .56], [MFD; *X* ^2^ = 0.38, *p* = .54], [FC; *X* ^2^ = 0.089, *p* = .77]), and body ([TFD; *X* ^2^ = 0.18, *p* = .66], [MFD; *X* ^2^ = 0.077, *p* = .78], [FC; *X* ^2^ = 0.77, *p* = .38]). However, during the second 10‐s viewing period, the autistic trait scores and gender affected fixations on the whole painting and the facial areas of the painting. Tables 4, 5, 6, 7 summarize the results of the influence of autistic trait scores and gender on TFD, MFD, and FC. The results indicated that an increase in autistic trait scores reduced TFD on the whole painting (intercept: estimate [Est.] = 7958.71, standard errors [SE] = 418.44, all units are in milliseconds) and faces (intercept: Est. = 1776.76, SE = 227.26) by 40.08 ± 21.5 (SE) and 26.16 ± 10.5 (SE), respectively, while men participants had longer TFD on faces by 411.98 ± 161.26 (SE). For FC (intercept: Est. = 4.99, SE = 0.59, all units are fixation number), higher autistic trait scores reduced FC by 0.067 ± 0.026 (SE). We also observed a significant effect of gender on FC, as men participants had more fixations on faces by 0.93 ± 0.4 (SE).

**TABLE 4:** Summary of the influence of autistic trait scores on eye‐tracking measures (the second 10‐s viewing period).

	TFD	MFD		FC		
	*X* ^2^	*p*	*X* ^2^	*p*	*X* ^2^	*p*
Whole painting	5.1	.024	0.094	.76	3.48	.062
AOI (face)	6.11	.013	0.064	.8	6.66	.0099
AOI (body)	0.17	.68	0.18	.67	0.0034	.95

Abbreviations: AOI, area of interest; FC, fixation count; MFD, mean fixation duration; Sig., significant; TFD, total fixation duration.

**TABLE 5:** Summary of the influence of gender (men) on eye‐tracking measures (the second 10‐s viewing period).

	TFD	MFD		FC		
	*X* ^2^	*p*	*X* ^2^	*p*	*X* ^2^	*p*
Whole painting	3.65	.056	0.33	.56	1.16	.28
AOI (face)	6.4	.011	1.89	.17	5.48	.019
AOI (body)	0.3	.58	0.16	.69	0.25	.62

Abbreviations: AOI, area of interest; FC, fixation count; MFD, mean fixation duration; Sig., significant; TFD, total fixation duration.

**TABLE 6:** Estimates and standard errors of the influence of autistic trait scores on eye‐tracking measures (the second 10‐s viewing period).

	TFD		FC	
	Est.	SE	Est.	SE
Whole painting	−40.08	21.5		
AOI (face)	−26.16	10.5	−0.067	0.026

Abbreviations: AOI, area of interest; Est., estimate; FC, fixation count; SE, standard error; TFD, total fixation duration.

**TABLE 7:** Estimates and standard errors of the influence of gender (men) on eye‐tracking measures (the second 10‐s viewing period).

	TFD		FC	
	Est.	SE	Est.	SE
AOI (face)	411.98	161.26	0.93	0.4

Abbreviations: AOI, area of interest; Est., estimate; FC, fixation count; SE, standard error; TFD, total fixation duration.

### Recall task

We performed a GLMM analysis to evaluate the fixed effects of the autistic trait scores and the participants' gender on the memory of paintings in the recall task. A complete model with the fixed effects of autistic trait scores and gender was constructed, and we assessed the significance of the fixed effects by comparing the reduced models to it. Random intercepts of paintings and participants were also included. The results show that autistic trait scores and gender did not affect the correctness of the response (autistic trait scores: *X* ^2^ = 0.021, *p* = .89; gender: *X* ^2^ = 0.0053, *p* = .94), and the average accuracy of judging whether the painting had been presented before in the free‐viewing task was very high: 88% ± 8.74% (*SE*).

## DISCUSSION

The present study assessed participants' visual attention when viewing figurative paintings and compared gaze behavior among participants according to their autistic trait scores. The results of the eye‐tracking data indicate that although autistic trait scores did not influence the eye‐tracking measures during the first 10 s of viewing the paintings, the gaze behavior differed according to the scores in the second 10 s. During the first 10 s, the TFD of an entire painting was not affected by autistic tendency; however, it was reduced by the autistic trait scores in the second 10 s. More importantly, higher scores correlated with fewer fixations and shorter durations on the face areas of the paintings. The fixation durations and number of fixations on the body parts of characters in a painting were not influenced by autistic trait scores. These results suggest that attention processes differ according to autistic tendencies. Participants with lower autistic tendencies fixated more and longer on the face areas of the paintings than those who scored higher on the AQ. When attending to social stimuli, such as faces, participants displayed different gaze behaviors according to their autistic trait scores, even when the stimuli with moderate ecological validity, such as paintings, were presented. Viewers' visual attention to body parts, however, did not differ among the participants. Moreover, men participants showed more fixations and longer fixation durations on the specific areas of the painting, that is, faces. In addition, the AQ scores did not affect performance on the recall task. All participants performed very well on the task, as the average percentage of correct responses was much higher (i.e., >88%) than the chance level.

Previous research has found reduced visual attention to faces in individuals with ASD in naturalistic contexts using photographs (Riby & Hancock, 2008, 2009). Consistent with this result, our study demonstrates that a semi‐naturalistic context, specifically the faces depicted in figurative paintings, also produces a similar trend. Reduced attention was found during the latter viewing period of the painting. At the beginning of the observation, no difference was found in gaze behavior among all participants. This result suggests that viewing patterns while looking at paintings are not random and may be modulated by the viewers' interest in social information. According to Chawarska et al. (2012), individuals with ASD may also find social stimuli too arousing. Consequently, they reduced their fixations on these areas.

The reduced interest in social cues during the latter viewing period suggests that the reason people with higher autistic tendencies were less drawn to faces may not be because of abnormalities in a specialized social attention process. This atypical socio‐attentional behavior may be accommodated in a general attentional process. The results are compatible with the findings of previous research that suggested that individuals with ASD have the same basic face bias (e.g., Shah et al., 2013). From a developmental perspective, domain specificity and uniqueness at a mechanistic level appear to lack unequivocal support, as there is no evidence to suggest that socio‐attentional behaviors are atypical in infants with emerging ASD (Elsabbagh et al., 2013). The domain‐general attention processes and nonattentional processes (e.g., social cognition or motivation) offer more plausible explanations for the reduced attention to social cues. For example, emotional information processing also plays a crucial role in attentional bias toward faces and scenes (Ghosn et al., 2019; Sahuquillo‐Leal et al., 2024). One study also found that gaze movements to complex social and nonsocial scenes are influenced by genetic factors (Kennedy et al., 2017).

Findings from drawings and cartographic images also supported the hypothesis of general attentional process. For example, the gaze behavior in terms of the fixation numbers and durations of both ASD and TD children was significantly higher and longer for cartoon‐like drawings of complex objects, than for basic ones, while no distinction was observed between the two groups (Kemner et al., 2007). Simplified drawings such as cartoons often use simplified shapes, colors, and designs to convey ideas and emotions quickly. Contrastingly, photographs generally maintain a consistent representation of the physical world, capturing visual details such as light, shadows, and textures with high fidelity. Hence, visual attention to social stimuli in realistic scenes may be reduced if differences in social attention are modulated in an endogenous domain‐general manner, as individuals with higher autistic traits may not retain their interest in visual details of social stimuli after completing the overview of the paintings.

Eye‐tracking research investigating viewing patterns in individuals with ASD has focused on real scenes that are complex and highly ecologically valid. Social stimuli presented in a form with moderate ecological validity between simple cartoon drawings and real photographs have been relatively less studied, for instance, figurative oil paintings. The paintings differ from real photographs, which record scenes directly as they exist in front of the camera. Although one study reported that children with ASD had different gaze patterns from TD children when viewing representative paintings, only two specific paintings were viewed by the participants (Zhang et al., 2022). They found that children with ASD and TD children had similar attention patterns when viewing a painting with only one character, but children with ASD looked for a significantly shorter amount of time at faces when two persons were shown in the painting. Visual attention to the entire painting also differed between ASD and TD children by the number of characters in the painting, as the FC of ASD children was around the same as that of TD children for the painting containing only one character, but significantly decreased when two persons were portrayed in one painting. In contrast, our study found no difference in fixation numbers on the whole painting if it had multiple characters, by using a large set of stimuli. It is possible that the effect was diminished because individuals with ASD pay attention differently according to the type of nonsocial objects. For example, they show decreased visual attention to particular stimuli, such as landscapes (Anderson et al., 2006). Unlike the paintings used in Zhang et al.'s study with an entire interior scene or a black background, our paintings had various backgrounds, including both interior and exterior scenes.

It is also important to examine the viewing behavior among Japanese people when looking at paintings to better understand the association between autistic traits and eye movements in cultural contexts. For years, the Western notions of “normality” seem to have dominated autism‐related discourses in autism‐related research, although the subjectivity inherent in what is considered “normal” may vary across cultures. Many behaviors are considered slightly exaggerated “normal” behaviors in Japanese society, while it is a complex challenge to comprehend neurodevelopmental conditions in non‐Western countries (Atherton et al., 2023). Gazing directly at faces is not traditionally encouraged among Japanese children (Morsbach, 1973), as it may be perceived as unpleasant and unapproachable. The gaze behavior, hence, may be influenced by cultural norms in a way that it is affected differently among different mediums according to ecological validity levels. In this study, we found that gaze patterns change over time in the free‐viewing task involving paintings, which is compatible with previous reports (Heidenreich & Turano, 2011; Zhou et al., 2024). Visual attention is significantly reduced by autistic trait scores only in the latter 10–20 s of viewing time. Therefore, gaze patterns of viewing paintings may be used as a potential biomarker, suggesting that reduced visual attention to social cues after acquiring the content of the painting may predict autistic tendencies. Among the general population, it has been suggested that the autistic population is male‐dominated and that men are more likely to exhibit autistic tendencies. However, girls may be at disproportionate risk of not receiving a clinical diagnosis, as the results of previous studies of reporting gender ratio varied significantly and substantially (Loomes et al., 2017). Surprisingly, our results suggest that gender did influence gaze behavior, but in a way that men participants are more likely to fixate longer on facial parts. In other words, men participants had more intensive visual attention to faces than women. This finding may also relate to cultural norms of gender roles in Japan, as Japanese society has gender‐specific expectations in social interactions. For example, men are often expected to be assertive, while women may be encouraged to be modest (Belarmino & Roberts, 2019).

In addition to the free‐viewing task, we included a recall task to investigate whether autistic traits influenced the memorability of the paintings. Kapsetaki and Zeki (2022) demonstrated that biological stimuli, especially faces, are more memorable. Thus, the memorability of a painting may be related to the visual attention to the faces in the painting. If individuals with higher autistic traits had reduced visual attention to the facial areas of the human characters, their memory of the paintings might be influenced. However, our results indicate that the memorability of paintings was not affected by autistic traits. Participants with higher autistic scores reduced their visual attention to faces after they had a sufficient amount of time (i.e., 10 s) to understand the content of the paintings. The paintings' content was acquired successfully, as their memorability was unaffected. Taken together, this result may also provide some support to the hypothesis that there is no specialized social attentional process within individuals with high autistic tendencies. A generalized attentional process for endogenous orienting with reduced interest in social cues seems more likely to explain the difference observed in social attention. Although the recall results did not show a difference in the memorability of the paintings according to autistic trait scores, further work is needed to determine the temporal constraints on the memorability of paintings among individuals with autistic traits.

Our study has some limitations. More work is required to reveal how gaze behavior differs in individuals with autistic traits or ASD when social information is presented in a medium with moderate ecological validity (e.g., paintings). First, although some participants scored higher than the threshold for potentially clinically significant autistic traits, the group mean autistic trait scores were relatively low. For this reason, our results may only reflect how gaze behavior varies according to a tendency toward ASD but are not indicative of individuals who are clinically diagnosed with ASD. The findings of this study should be validated with pre‐screened participants who are diagnosed with ASD. Second, the gender distribution of this study is not consistent with the gender distribution of autism and may not accurately describe the characteristics of the male‐dominated autistic population. The influence of gender on how autistic traits influence gaze behavior using a data set with a normalized gender distribution should be addressed in future research. Moreover, future studies should investigate how the social intensities of paintings vary according to the number of characters and situations and how such factors or nonsocial information influence visual attention in social scenes among individuals with autistic traits or with ASD. Finally, the study should be repeated considering the influence of participants' intelligence on visual attention when viewing paintings by measuring their baseline ability (i.e., intelligence levels).

In summary, this study explored whether the visual attention of people with higher autistic trait scores differed from those with lower scores on the AQ. Gaze behavior was assessed when the participants viewed figurative paintings with moderate ecological validity. The eye‐tracking results demonstrate that the higher the autistic trait score, the lower the number of fixations and the shorter the fixation duration on the face areas of the paintings. No significant difference was found in visual attention to the entire painting according to the autistic trait scores. For the first time, we found that an association may exist between autistic trait scores and gaze behavior during a 20‐s presentation time using a large set of figurative paintings. This finding helps to explore the specific circumstances in which gaze behavior may differ during a human‐unique experience, the experience of art.

## CONFLICT OF INTEREST STATEMENT

The authors declare no conflicts of interest.

## ETHICS STATEMENT

This study was approved by the Ethics Committee of Keio University (approval number: 22016) and conducted in accordance with the ethical standards in the 2013 Declaration of Helsinki.

## Supporting information


**Data S1.** Supporting Information.

**Data S2.** Supporting Information.

**Data S3.** Supporting Information.

**Table S1.** List of figurative paintings.

**Table S2.** The autism‐spectrum quotient (AQ) scores of each participant.
