# Supplementary material for: Gaze behavior when looking at paintings may predict autistic traits
Source: Psych J. 2025 Jan 6;14(2):267–76. doi: 10.1002/pchj.810 (PMC11961244; doi:10.1002/pchj.810)
Supplement: Supplementary file 4 — Table S1. List of figurative paintings. [file PCHJ-14-267-s004.docx]

Table S1

| **List of figurative paintings** |  | |  |  |
| --- | --- | --- | --- | --- |
| Title | | Artist | | No |
| Il Giornale | | Alan Feltus | | 1 |
| Carnevale-1923 | | Antonio Donghi | | 2 |
| The Corduroy Road | | Art Rosenbaum | | 3 |
| Calling of Saint Matthew | | Caravaggio | | 4 |
| Las Meninas | | Diego Velazquez | | 5 |
| Domenico Beccafumi | | Die Strafe Des Höllenfeuers | | 6 |
| The Ballet Class | | Edgar Degas | | 7 |
| The Balcony | | Edouard Manet | | 8 |
| Nighthawks | | Edward Hopper | | 9 |
| Berlin Street Scene | | Ernst Ludwig Kirchner | | 10 |
| Christ on the Sea of Galilee | | Eugène Delacroix | | 11 |
| Transfiguration | | F. Scott Hess | | 12 |
| Church Attendance in the Spring | | Ferdinand Georg Waldmüller | | 13 |
| Trouville | | Fernand Leger | | 14 |
| Annunciation | | Fra Angelico | | 15 |
| The Bashful Cousin | | Francis William Edmonds | | 16 |
| The Third of May 1808 (Execution of the Defenders of Madrid) | | Francisco Goya | | 17 |
| Madonna with Child and Two Donors | | Gentile Bellini | | 18 |
| The Cheat with the Ace of Diamonds | | Georges de la Tour | | 19 |
| Sunday Afternoon on the Island of La Grande Jatte | | Georges Seurat_ | | 20 |
| Paris Street, Rainy Day | | Gustave Caillebotte | | 21 |
| The Wheat Sifters | | Gustave Courbet | | 22 |
| Moorish Screen | | Henri Matisse | | 23 |
| War or the Ride of Discord | | Henri Rousseau | | 24 |
| Unexpected Visitors | | Ilya Repin | | 25 |
| The Crucifixion | | Jacopo Bellini | | 26 |
| The Oath of Horatii | | Jacques-Louis David | | 27 |
| Dancing Lesson | | Jan Steen | | 28 |
| Death of Leonardo da Vinci | | Jean Auguste Dominique Ingres | | 29 |
| Broken Eggs | | Jean-Baptiste Greuze | | 30 |
| The Gleaners | | Jean-Francois Millet | | 31 |
| The Procuress | | Johannes Vermeer | | 32 |
| An Experiment on a Bird in the Air Pump | | Joseph Wright | | 33 |
| Boys on the Background of the City | | Kuzma Petrov-Vodkin | | 34 |
| The Betrothed and Eiffel Tower | | Marc Chagall | | 35 |
| Red Poppies | | Mary Cassatt | | 36 |
| Guernica | | Pablo Picasso | | 37 |
| The Card Players | | Paul Cezanne | | 38 |
| The Dance | | Paula Rego | | 39 |
| The Death of Hippolytus | | Peter Paul Rubens | | 40 |
| The Luncheon of the Boating Party | | Pierre-Auguste Renoir | | 41 |
| Peasant Wedding | | Pieter Bruegel the Elder | | 42 |
| Lady at Her Toilette | | Pietro Longhi | | 43 |
| Madonna del Prato | | Raphael | | 44 |
| The Nightwatch | | Rembrandt | | 45 |
| Deposition | | Rogier van der Weyden | | 46 |
| The Spring | | Sandro Botticelli | | 47 |
| “Again you found me” (Carter and Luca) | | Sara Chess | | 48 |
| The Night Cafe | | Vincent van Gogh | | 49 |
| The Marriage Contract | | William Hogarth | | 50 |
