# Supplementary material for: Gaze behavior when looking at paintings may predict autistic traits
Source: Psych J. 2025 Jan 6;14(2):267–76. doi: 10.1002/pchj.810 (PMC11961244; doi:10.1002/pchj.810)
Supplement: Supplementary file 5 — Table S2. The autism‐spectrum quotient (AQ) scores of each participant. [file PCHJ-14-267-s005.docx]

Table S2

**The autism-spectrum quotient (AQ) scores of each participant**

| Pid | AQ score |
| --- | --- |
| p1 | 26 |
| p2 | 14 |
| p3 | 10 |
| p4 | 20 |
| p5 | 23 |
| p6 | 23 |
| p7 | 19 |
| p8 | 16 |
| p9 | 23 |
| p10 | 12 |
| p11 | 25 |
| p12 | 15 |
| p13 | 17 |
| p14 | 15 |
| p15 | 16 |
| p16 | 11 |
| p17 | 14 |
| p18 | 29 |
| p19 | 12 |
| p20 | 10 |
| p21 | 12 |
| p22 | 35 |
| p23 | 15 |
| p24 | 14 |
| p25 | 33 |
| p26 | 17 |
| p27 | 10 |
| p28 | 26 |
| p29 | 33 |
| p30 | 16 |
| p31 | 25 |
| p32 | 13 |
| p33 | 12 |
| p34 | 8 |
| p35 | 27 |
| p36 | 23 |
| p37 | 28 |
| p38 | 9 |
| p39 | 17 |
| p40 | 22 |
| p41 | 13 |
| p42 | 22 |
| p43 | 17 |
| p44 | 14 |
| p45 | 10 |
| p46 | 15 |
